# Suppressive effect of TNF-α and IL-1 on alveolar fibroblast proliferation in sarcoidosis

**DOI:** 10.1155/S0962935192000474

**Published:** 1992

**Authors:** E. Fireman, D. Aderka, S. Ben Efraim, J. Greif, D. Wallach, M. Topilsky

**Affiliations:** 1 Department of Pulmonary Diseases and Allergic Diseases TeI-Aviv Medical Center Ichilov Hospital 6 Weizmann Street TeI-Aviv 64268 Israel; 2 Department of Internal Medicine “T” Ichilov Hospital 6 Weizmann Street TeI-Aviv 64239 Israel; 3 Department of Human Microbiology Sackler School of Medicine TeI-Aviv University Ramat Aviv TeI-Aviv 69978 Israel; 4 Department of Virology Weizmann Institute of Science Rehovot 76326 Israel

## Abstract

The nature of soluble factors that regulate fibroblast proliferation have not been finally characterized. Our aim was to study the role of tumour necrosis factor α (TNF-α) and interleukin-1 (IL-1) in the suppressive activity of alveolar macrophages on autologous lung fibroblasts proliferation in sarcoidosis. We found that supernatants recovered from alveolar macrophages suppressed the proliferation of alveolar fibroblast in sarcoidosis by 35.5 ± 1.13% compared to 3 ± 16% in controls (p < 0.001 between the two groups). This suppression correlated with high content of TNF-α and IL-1 in sarcoidosis patients stage II-III (7.7 ± 2.9 ng/ml TNF-α and 157 ± 53 U/ml IL-1 compared to 3.4 ± 2.4 ng/ml TNF-α and 43 U/ml IL-1 in controls; p < 0.01 and p < 0.001, respectively). Both cytokines in sarcoidosis stage I were within the normal ranges. Exogenous TNF-α (1000-0.5 ng/ml) and IL-1 (500-0.24 ng/ml) had an additive suppressive activity on fibroblast proliferation which was partially reversed by indomethacin.


Mediators of Inflammation, 1, 319–322 (1992)

### Suppressive effect of TNF-$\alpha$ and IL-1 on alveolar fibroblast proliferation in sarcoidosis

E. Fireman,[1,CA] D. Aderka,[2] S. Ben Efraim,[3] J. Greif,[1] D. Wallach[4] and M. Topilsky[1]

[1] Department of Pulmonary Diseases and Allergic Diseases Tel-Aviv Medical Center, Ichilov Hospital, 6 Weizmann Street, Tel-Aviv 64268, Israel;
[2] Department of Internal Medicine "T", Ichilov Hospital, 6 Weizmann Street, 64239 Tel-Aviv, Israel;
[3] Department of Human Microbiology, Sackler School of Medicine, Tel-Aviv University, Ramat Aviv, Tel-Aviv 69978 Israel;
[4] Department of Virology, Weizmann Institute of Science, Rehovot, 76326 Israel

[CA] Corresponding Author

**THE nature of soluble factors that regulate fibroblast proliferation have not been finally characterized. Our aim was to study the role of tumour necrosis factor $\alpha$ (TNF-$\alpha$) and interleukin-1 (IL-1) in the suppressive activity of alveolar macrophages on autologous lung fibroblasts proliferation in sarcoidosis. We found that supernatants recovered from alveolar macrophages suppressed the proliferation of alveolar fibroblast in sarcoidosis by 35.5 $\pm$ 1.13% compared to 3 $\pm$ 16% in controls ($p < 0.001$ between the two groups). This suppression correlated with high content of TNF-$\alpha$ and IL-1 in sarcoidosis patients stage II–III (7.7 $\pm$ 2.9 ng/ml TNF-$\alpha$ and 157 $\pm$ 53 U/ml IL-1 compared to 3.4 $\pm$ 2.4 ng/ml TNF-$\alpha$ and 43 U/ml IL-1 in controls; $p < 0.01$ and $p < 0.001$, respectively). Both cytokines in sarcoidosis stage I were within the normal ranges. Exogenous TNF-$\alpha$ (1000–0.5 ng/ml) and IL-1 (500–0.24 ng/ml) had an additive suppressive activity on fibroblast proliferation which was partially reversed by indomethacin.**

**Key words:** Alveolar fibroblasts, Alveolar macrophages, Indomethacin, Interleukin-1, $PGE_2$, Sarcoidosis, Tumour necrosis factor $\alpha$

### Introduction

The injury of any tissue, whether caused by trauma, microbes or a foreign antigen, initiates any inflammatory response which leads to the normal physiological process of healing. Acute pneumonias, even those with marked necrosis, can heal without excessive scarring. In contrast, interstitial disorders may result in excessive fibrosis with loss of organ function. The processes responsible for these different outcomes need further definition.

Mononuclear cells are important regulators of the fibrotic response. Their regulatory effects are at least partially mediated by soluble factors that can stimulate[1,2] or inhibit[3,4] fibroblast proliferation.

Interleukin-1 (IL-1) protein has pronounced effects on various lineages of cells and other cells of mesenchymal origin.[5,6] Stimulated monocytes and macrophages also elaborate tumour necrosis factor $\alpha$ (TNF-$\alpha$) which is now known to have a broad range of cytoregulatory effects[7,8] including the ability to regulate cell proliferation. Moreover TNF-$\alpha$ and IL-1 synergistically stimulate $PGE_2$ elaboration by confluent fibroblast.[9] Because mononuclear cell inflammation precedes the fibrotic stage in sarcoidosis the cytokines released by alveolar macrophages and their role on fibroblast growth may have a crucial role. To test this theory we determined the suppressive effect of alveolar macrophage supernatants from patients with sarcoidosis on proliferation of autologous alveolar fibroblasts and correlated it with their TNF-$\alpha$ and IL-1 content.

### Materials and Methods

*Study population:* Patients were subdivided into three groups. Sarcoidosis patients were diagnosed by clinical and roentgenological presentation, a positive Kweim test or positive biopsy of non-caseating granuloma. According to the X-rays these patients were grouped into stage I (four untreated patients) and stage II–III (nine untreated patients). For controls, seven untreated patients undergoing bronchoscopy due to unexplained persistent cough or after an episode of mild haemoptysis. All of them had chest roentgenograms within normal limits. Written informed consent was obtained from each subject before bronchoscopy and bronchoalveolar lavage.

*Bronchoalveolar lavage:* After informed consent, bronchoscopy with bronchoalveolar lavage was performed with a flexible fibre optic bronchoscope (Olympus BF-B2) as previously described.[10]

*Preparation of alveolar macrophages:* The recovered fluid was collected in specimen traps, filtered through sterile gauze and centrifuged at 400 × $g$ for 15 min at 4°C. The pellet obtained was washed three times with cold PBS (Biological Industries, Beit Haemek), the number of viable cells was counted and purified by adherence as previously described.[10]

© 1992 Rapid Communications of Oxford Ltd

*Preparation of lung fibroblasts:* Alveolar fibroblasts (Afb) were obtained from bronchoalveolar lavage cells after long-term incubation (3–4 weeks) as previously described.[11] Control fibroblasts were derived from histologically normal areas of lungs resected for diagnostic reasons. The techniques of preparation and the proliferative characteristics of these cells have been described.[12]

*Preparation of alveolar macrophage supernatants:* Supernatants were obtained from alveolar cells cultured as previously described. The cells were allowed to adhere for 1 h, washed vigorously and overlayed by an identical volume of complete RPMI medium (2% foetal calf serum (FCS), antibiotic–antimycotic) with or without 10 μg/ml lipopolysaccharide (LPS) (Difco, St Louis, USA 055:B55). The cells were incubated for 24 h in 5% $CO_2$. Aliquots of the medium harvested from cultures were centrifuged, filtered and frozen for future use at −70°C.

*Fibroblast proliferation test:* Fibroblast suspension (100 μl) was recovered as described previously.[10] Briefly, cells were washed and resuspended in Dulbecco modified Eagles medium (DMEM) with 1% FCS, 2-mercaptoethanol ($5 \times 10^{-5}$ M) and 1% antibiotic–antimycotic mixture at $10^5$ cells/ml. Fibroblasts were dispensed into each well of 96-well flat-bottomed microtitre plates and allowed to attach for 1–2 h. Aliquots (100 μl) of supernatants of LPS pulsed alveolar macrophages were added. Cultures were incubated in a humidified 5% $CO_2$ atmosphere for 72 h, and pulsed with 1 μCi$^3$H-thymidine for the last 4 h of culture. For harvesting, the supernatant from each well was aspirated and 0.1 ml trypsin–EDTA was added to each well. Detached cells were harvested and counted. The growth of fibroblasts in LPS stimulated alveolar macrophage supernatant or cytokines (rIL-1 500–0.24 ng/ml — Glaxo IMB; TNF-α 1000–0.5 ng/ml — Genentech Inc, San Francisco, CA, USA) was compared with the growth of fibroblasts in complete DMEM with and without a final concentration of 10 μg/ml LPS. Neutralization of TNF-α and IL-1 activity was done by anti-TNF and anti-IL-1 MoAbs (240 μg/ml, Genentech and 20 μg/ml, Genzime respectively) and $PGE_2$ was reversed by indomethacin (1 μg/ml, Sigma, Chemical Co, St Louis Mo, USA).

*Assay of prostaglandin, TNFα and IL-1 production:* Aliquots of alveolar macrophage supernatants (24 h production) were assayed for $PGE_2$ and IL-1 production. $PGE_2$ was assayed using an ELISA kit (Advanced Magnetic Inc.), TNF-α was measured by a biological assay on A9 target cells, and IL-1 by the C3H/HeJ thymocyte comitogenic assay, as described previously.[13]

**Table 1.** TNF-α, IL-1 and $PGE_2$ mean contents of alveolar macrophage supernatants*

| Diagnosis | TNF-α (ng/ml) | IL-1 (U/ml) | $PGE_2$ (ng/ml) |
|---|---|---|---|
| Stage I (4)^ | 1.1 ± 0.8 | 42 ± 35 | 0.13 ± 0.05 |
| Stage II–III(9) | **7.7 ± 2.9 | +157 ± 33 | ++0.45 ± 0.28 |
| Control (7) | 3.4 ± 2.4 | 43 ± 26 | 0.34 ± 0.2 |

^No. of cases.
* TNF and IL-1 were measured by biological methods in 24 h supernatants of six stimulated alveolar macrophages. $PGE_2$ was measured by an ELISA assay in 72 h supernatants of six stimulated alveolar macrophages.
** $p < 0.01$ compared to controls.
+ $p < 0.001$ compared to controls.
++ $p < 0.001$ compared to stage I sarcoidosis; no significant differences between $PGE_2$.
Content of sarcoidosis stage II–III and control group.

### Results

The content of TNF-α, IL-1 and $PGE_2$ in alveolar macrophage supernatants in all patients tested is shown in Table 1. No differences were observed in the secretion of the compounds between sarcoidosis stage I and the control group. A high secretion of IL-1 and TNF-α not correlated to increase in secretion of $PGE_2$ is seen in supernatants of sarcoidosis patients stage II–III. The effects of these supernatants were tested on the proliferation of alveolar fibroblasts (Table 2). Alveolar macrophage supernatants from sarcoidosis patients suppressed the proliferation of alveolar fibroblasts by 38 ± 7.13% whereas alveolar macrophage supernatants from controls induced only a slight suppression of 3 ± 16% ($p < 0.01$ between sarcoidosis patients and controls).

The suppressive activity of alveolar macrophages from stage II–III sarcoidosis patients was correlated with a marked increase in secretion of IL-1 and TNF-α. In view of this fact we determined if exogenous cell-free rTNF-α and IL-1 have suppressive effects on fibroblasts. As shown in Table 3 and Fig. 1 both IL-1 and TNF-α suppressed fibroblast proliferation. The suppressive effect of IL-1 was reversed by indomethacin (Table 3). The specificity of suppressive activity of TNF-α and IL-1 was ascertained by neutralization with anti-TNF-α and anti-IL-1 mAbs (Table 3). Concomitant addition of IL-1 and TNF-α resulted in an additive suppressive effect (Fig. 2) which was also partially reversed by indomethacin.

### Discussion

The cells involved in the inflammatory response generate a variety of factors which appear to regulate the healing process through the recruitment, stimulation of growth, and matrix synthesis

**Table 2.** Effect of alveolar macrophage supernatants on alveolar fibroblast proliferation

| + Medium | Proliferation of sarcoid alveolar fibroblasts* | | | |
|---|---|---|---|---|
| | + Sup. of sarcoidosis alveolar macrophages | % Supp/enhan.⁺ | + Sup. of control alveolar macrophages | % Supp/enhan.⁺⁺ |
| 7753 ± 1007 | 4940 ± 506 | 37 | 7001 ± 805 | 10 |
| 9399 ± 297 | 5442 ± 516 | 42 | 12213 ± 1462 | −29 |
| 1801 ± 1007 | 921 ± 60 | 49 | 1441 ± 210 | 20 |
| 7613 ± 1007 | 4971 ± 1661 | 35 | 8153 ± 574 | −7 |
| 6047 ± 268 | 4507 ± 315 | 26 | 5630 ± 162 | 7 |
| 2633 ± 853 | 1528 ± 113 | 42 | 2200 ± 193 | 13 |

* One hundred μl of autologous alveolar fibroblasts at a final concentration of $10^6$ cell/ml were incubated in 96-well microplates with or without AM supernatants for 72 h. For the last 4 h cells were pulsed with 0.1 μCi[$^3$H]-thymidine.
⁺ Mean suppression by sarcoidosis alveolar macrophages supernatants 38.5 ± 7.13%.
⁺⁺ Mean suppression by control alveolar macrophages supernatants. 3 ± 16%. $p < 0.001$ between two groups.
+ means suppression; − means enhancement.

**Table 3.** Effect of indomethacin and MoAbs to the induced IL-1 and TNF depression on fibroblast proliferation*

| | Sarcoidosis | Controls |
|---|---|---|
| Medium | 4778 ± 235 | 54173 ± 1327 |
| IL-1⁺ | 3561 ± 607 | 47808 ± 1866 |
| IL-1 + Ind⁺⁺ | 7516 ± 354 | 48977 ± 9649 |
| IL-1 + mAbs^ | 5508 ± 142 | 46689 ± 780 |
| Medium | 9968 ± 292 | 60069 ± 28 |
| TNF⁺ | 6704 ± 1486 | 22134 ± 853 |
| TNF + Ind⁺⁺ | 5520 ± 1577 | 31018 ± 2006 |
| TNF + mAbs^ | 18714 ± 3145 | 44152 ± 1530 |

* cpm of $^3$[H]-thymidine incorporation of fibroblasts.

⁺ IL-1 = 15 ng/ml; TNF = 10 ng/ml.

⁺⁺ Indomethacin = 1 μg/ml.

^mAbs IL-1 = 20 μg/ml; mAbs TNF = 240 μg/ml.

by connective tissue cells.[14] Accumulating evidence indicates that monocyte–macrophage products play an important role in modulating connective tissue alteration in inflammatory diseases.[15] Recent studies have focused on the role of IL-1, TNF-α, IFNτ,[16–18] IL-6[19] and $PGE_2$[20] in the regulation of fibroblast growth and function in the normal lung but little is known about the role of these factors in interstitial lung diseases.

To understand further the role of these factors we characterized the effect of alveolar macrophage supernatants from patients with sarcoidosis on alveolar fibroblast proliferation and compared it with the control group. The experiments showed that sarcoidosis supernatants exerted an inhibitory effect whereas the control supernatants induced only a slight suppression or enhancement. These results confirmed those previously shown[21] but they reflect more closely the *in vivo* situation as each alveolar macrophage supernatant was incubated with the autologous fibroblasts.

Although previous studies showed already that alveolar macrophages from sarcoidosis patients secrete increased amounts of IL-1[22] and TNF-α,[23] the importance of the intercytokine interactions in correlation with different staging in sarcoidosis has not been investigated adequately. We demonstrate here that alveolar macrophages secrete high amounts of TNF and IL-1 and also exhibit marked

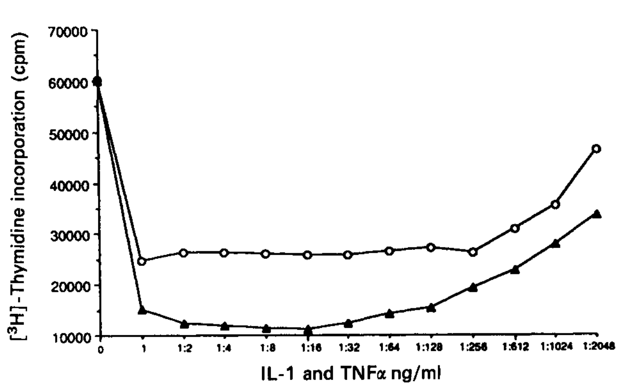

FIG. 1. Effect of IL-1 and TNF-α on fibroblast proliferation. Combined rIL-1 in serial dilutions of 500–0.24 ng/ml and TNF-α in serial dilutions of 1000–0.5 ng/ml were added to control fibroblasts at a final concentration of $10^5$ cells/ml for 72 h. During the last 4 h cells were pulsed with 1 μCi [$^3$H]-thymidine and incorporation was expressed in counts per minute (cpm). $p < 0.01$ between baseline of fibroblasts and after addition of TNF (1:128), and $p < 0.001$ after addition of TNF and IL-1. —▲—, IL-1 + TNF-α; —○—, IL-1 + TNF-α + IND.

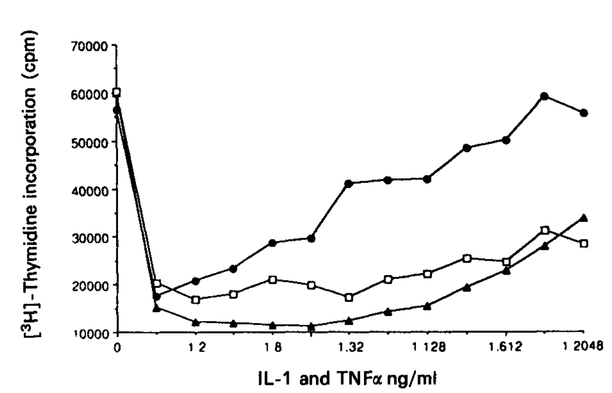

FIG. 2. Effect of indomethacin on the TNF-α and IL-1 induced suppression of fibroblasts. Proliferation of control fibroblasts was tested in the presence of TNF-α and IL-1 with and without 1 μg/ml indomethacin. During the last 4 h cells were pulsed with 1 μCi[$^3$H]-thymidine and incorporation was expressed in counts per minute (cpm). $p < 0.001$ between fibroblast proliferation with TNF-α and IL-1 (1:128) before and after addition of indomethacin. —▲—, IL-1 + TNF-α; —□—, TNF-α; —●—, IL-1.

suppressive activity of fibroblast proliferation only in patients with stage II–III sarcoidosis. The high secretion of TNF-α and IL-1 was not correlated to similar increases in $PGE_2$ secretion. It should be noted that down-regulation of $PGE_2$ secretion by alveolar macrophages from sarcoidosis patients has been reported already.[24]

The high secretion of both cytokines seems to have a definitive suppressive role as the exogenous addition of TNF-α and IL-1 have a net suppressive effect on fibroblast proliferation within the range secreted by alveolar macrophages in sarcoidosis patients (1–10 ng/ml for TNF and 100–500 U/ml for IL-1).

Sarcoidosis is a multi-system granulomatous disease with a benign clinical course in the majority of the patients. The disease progresses from granulomatous inflammation to fibrosis only in about 10–20% of cases.[25,26] It is possible that the high secretion of TNF-α and IL-1 is involved in limiting the fibrotic response. In stage I–II sarcoidosis we showed high secretion of IL-1 only, which can have simultaneously stimulatory and inhibitory effects on fibroblast proliferation as already reported for osteoclasts.[27] It is of interest that indomethacin partially reversed the suppressive effect of TNF-α and IL-1. Apparently this effect is not due to the well-known property of indomethacin as a cyclooxygenase inhibitor but may reflect a direct effect of cytokines.

These studies show that cytokines can have multiple effects on fibroblast proliferation and that the effect that is noted depends on the entire set of regulatory factors affecting the target cell.

### References

1. De Lustro F, Sherer G, Leroe E. Human monocyte stimulation of fibroblasts growth by soluble mediator(s). *J Reticulendothel Soc* 1980; **28:** 519–525.
2. Bitterman PB, Rennard SI, Hunninghake GW, Crystal RG. Human alveolar macrophages growth factor for fibroblasts: regulation and partial characterization. *J Clin Invest* 1982; **70:** 806–822.
3. Elias JA, Rossman MD, Zurier RB, Daniel RP. Human alveolar macrophages inhibition of lung fibroblast growth: a prostaglandin-dependent process. *Am Rev Respir Dis* 1985; **131:** 94–99.
4. Clark JG, Kostal KM, Marino BA. Bleomycin induced pulmonary fibrosis in hamsters: and alveolar macrophage product increases fibroblast growth relationship to fibroblast prostaglandin production and density defined monocyte subpopulation. *J Leuk Biol* 1985; **37:** 15–28.
5. Oppenheim JJ, Mizel SB, Melzer MS. Comparison of lymphocyte and mononuclear phagocyte derived mitogenic "amplification" factors. In: Cohen S, Pick E, Oppenheim JJ, eds. *Biology of Lymphokines.* New York: Academic Press, 1979; 291–323.
6. Rupp EA, Cameron PM, Ranawat CS, Schmidt JA, Bayne EK. Specific bioactivities of monocyte derived interleukin 1α and interleukin 1β are similar to each other on cultured murine thymocytes and on cultured human connective tissue. *J Immunol* 1986; **78:** 836–839.
7. Sugarman BJ, Aggarwal BB, Hass PE, Fibari IS, Palladino MA Jr, Shepard HM. Recombinant human tumor necrosis factor α effects on proliferation of normal and transformed cells *in vitro. Science* 1985; **230:** 943–945.
8. Dayer JM, Bentler B, Cerami A. Cachectin/tumor necrosis factor stimulates collagenase and prostaglandin E production by human synovial cells in dermal fibroblasts. *J Exp Med* 1985; **162:** 2163–2168.
9. Elias JA, Gustilo K, Balder W, Freundlich B. Synergistic stimulation of fibroblast prostaglandin production by recombinant interleukin 1 and tumor necrosis factor. *J Immunol* 1987; **138:** 3812–3816.
10. Fireman EM, Ben Efraim S, Greif J, Kivity S, Topilsky RM. Suppressor cell activity of human alveolar macrophages in interstitial lung diseases. *Clin Exp Immunol* 1988; **73:** 111–116.
11. Fireman E, Ben Efraim S, Messer Y, Dabush S, Greif J, Topilsky M. Cell-free supernatant of sarcoid alveolar macrophages suppress proliferation of sarcoid alveolar fibroblasts. *J Clin Immunol Immunopathol* 1991; **59:** 368–378.
12. Bitterman PB, Wewers MD, Rennard SI, Adelberg S, Crystal RG. Modulation of alveolar macrophage-driven fibroblast proliferation by alternative macrophage mediators. *J Clin Invest* 1986; **77:** 700–708.
13. Fireman EM, Ben Efraim S, Greif J, Alguetti D, Ayalon D, Topilsky M. Suppressive activity of alveolar macrophages and blood monocytes from interstitial lung diseases: role of released soluble factors. *Int J Immunopharmacol* 1989; **7:** 751–760.
14. Angelli M, Wahl SM. Cytokines and fibrosis. *Clin Exp Rheumatol* 1986; **4:** 379–388.
15. Le-Roy EC, Trojanowska MI, Smith EA. Cytokines and human fibrosis. In: Bienvenu J, Fradelizi D, eds. *Cytokines and Inflammation.* Paris: John Libbey Eurotext, 1991; pp 141–150.
16. Elias JA, Gustilo K, Freundlich B. Human alveolar macrophage and blood monocyte inhibition of fibroblasts proliferation: evidence for synergy between IL-1 and Tumor Necrosis Factor. *Am Rev Respir Dis* 1988; **138:** 1595–1603.
17. Elias JA. TNF interacts with IL-1 and IFN to inhibit fibroblast proliferation via fibroblast dependent and independent mechanism. *Am Rev Respir Dis* 1988; **138:** 652–658.
18. Elias JA, Jimenez SA, Freundlich B. Recombinant σ, α, β, interferon regulation of human lung fibroblast proliferation. *Am Rev Respir Dis* 1987; **135:** 62–65.
19. Elias JA, Trinchieri G, Beck JM, *et al.* A synergistic interaction of IL-6 and IL-1 mediates the thymocyte-stimulating activity produced by recombinant IL-1 stimulated fibroblasts. *J Immunol* 1989; **142:** 509–514.
20. Elias JA, Rossman MD, Zurier RB, Daniele RP. Human alveolar macrophage inhibition of lung fibroblast growth. *Am Rev Respir Dis* 1985; **131:** 94–99.
21. Elias JA, Rossman MD, Daniele RP. Blood and lung mononuclear cell inhibition of fibroblast growth in sarcoidosis. *Am Rev Respir Dis* 1984; **130:** 1050–1057.
22. Hunnighake GW. Release of interleukin-1 by alveolar macrophages of patients with active pulmonary sarcoidosis. *Am Rev Respir Dis* 1984; **129:** 569–572.
23. Bachwich PR, Lynch JP III, Wiggins R, Kunkel SL. Tumor necrosis factor-like activity in normal and sarcoid alveolar macrophages (abstract). *Am Rev Respir Dis* 1986; **133:** (Suppl A244).
24. Bachwich PR, Lynch JP, Kunkel SL. Arachidonic acid metabolism is altered in sarcoid alveolar macrophages. *Clin Immunol Immunopathol* 1987; **42:** 27–37.
25. Crystal RG, Roberts WC, Hunnighake GW, Gadek JE, Fulmer JD, Line BR. Pulmonary sarcoidosis: a disease characterized and perpetuated by activated lung T lymphocytes. *Ann Intern Med* 1981; **94:** 73–94.
26. Thrasher DR, Briggs DD. Pulmonary sarcoidosis. *Clin Chest Med* 1982; **3:** 569–572.
27. Gowen M, Wood DD, Grahm R, Russell GG. Stimulation of the proliferation of human bone cells *in vivo* by human monocyte products with interleukin-1 activity. *J Clin Invest* 1985; **75:** 1223–1229.

ACKNOWLEDGEMENTS. This work was drafted when one of us (S.B.E.) was on sabbatical leave at the Erasmus University, Rotterdam. The stay in Rotterdam was supported by a research fund raised by 'Supporters of the Joint Dutch–Israeli Medical Research' under the auspices of the Israeli Cancer Association, Tel-Aviv, Israel and the Erasmus University Foundation. S.B.E. is a fellow of the Lautenberg Center for General and Tumor Immunology, Hadassah Medical School, The Hebrew University, Jerusalem, Israel.

**Received 12 May 1992; accepted in revised form 28 July 1992**
